# Global child health in higher education in Germany: a mixed-methods study

**DOI:** 10.1080/16549716.2022.2093464

**Published:** 2022-08-15

**Authors:** Dennis Küppers, Michael Galatsch, Ralf Weigel

**Affiliations:** a Paediatric Heart Center, University Hospital Giessen and Marburg, Justus-Liebig-University, Giessen, Germany; b Friede-Springer Endowed Professorship for Global Child Health, Faculty of Health, School of Medicine, Witten/Herdecke University, Witten, Germany

**Keywords:** Child, paediatric, global health, higher education, Germany

## Abstract

**Background:**

Germany has an ambitious global health strategy, yet its universities provide few opportunities for global child health researchers. Improved understanding of the reasons and the academic role of global child health is needed.

**Objective:**

The objective of this study is to offer insights into Germany’s academic global child health landscape by describing the actors and their priorities in research and education and by analysing perceived barriers and opportunities.

**Methods:**

We used a sequential exploratory mixed-method design. Participants were selected purposively to represent German global child health academics. Information was gathered first from a 33-item online survey and from interviews conducted four to six months post-survey. Surveys were analysed descriptively. A joint thematic approach using content analysis was used to analyse interview transcripts.

**Results:**

Four categories emerged: training and professional orientation; professional realities; representation and advocacy, and barriers. Of the 20 survey participants (median [IQR] age 55 years [17], five female), seven agreed to be interviewed. Research experiences abroad shaped individuals’ career choices in global child health. They engaged in global child health education, primary health care and access to health services, frequently in clinical and humanitarian settings, but spent little time on global child health-related activities. Participants were active and valued in international networks and keen to extend their activities. Yet they felt under-represented academically and reported multiple structural and individual barriers in Germany. They perceived a lack of leadership positions, career paths, funding opportunities, and institutional and project support which limits academic advancement.

**Conclusions:**

Germany’s global child health experts are motivated to engage with global child health-related topics but face difficulties in advancing academically. Academic actors may need to intensify research and training efforts in order to expand global child health’s scientific base in Germany.

## Background

Universities must analyse and provide solutions for global health challenges through research and education, especially when times are difficult [1]. Global-governing bodies such as the World Health Organisation (WHO) and the United Nations International Children’s Emergency Fund (UNICEF) depend on evidence and expertise stemming from academic debates because evidence will inform WHO’s policies and their implementation. The new German global health strategy realises this dependency, and aims to ‘promote the deployment of German experts and young professionals in international health organisations and bodies’ [2]. In order to achieve this, public and global health scientists advocate for global health academic chairs at German universities. This will strengthen global health research and foster scholarship leading to meaningful scientific contributions to the academic global health debate [3]. However, Germany’s universities remain ill-prepared because global health research and teaching capacity and career opportunities for young scientists in the field are still lacking [4,5].

This imbalance between the aspirations outlined in Germany’s global health strategy and poorly developed academic foundations is particularly striking in global child health (GCH). The global dimension of child health today and the multiple risks to children’s health are apparent. They undermine the gains achieved for child health globally [6]. The Sars-CoV-2 pandemic directly and indirectly affects children’s health and well-being [7,8]. The changing climate with more frequent extreme weather events, changing patterns of vector-borne diseases, and adverse impacts on food production and distribution caused by industrialised countries over decades, has negative consequences for children and their families, especially in poorer countries [9,10]. Furthermore, social conditions such as poor housing, a lack of nurturing care and education as well as promotion of unhealthy products and choices by the private sector harm children’s health [11].

High-income countries, such as Germany, are particularly responsible for children’s health and well-being. They should do more to place children in the centre of the Sustainable Development Goals [12], to end preventable deaths and create enabling environments so that every child can reach its full potential [13]. Recently, the WHO and UNICEF launched an initiative to re-design child health programmes to provide the tools to realise the global strategy for women’s, children’s and adolescents’ health [6]. However, Germany’s contributions to this initiative and many other global child health initiatives are mostly absent – a situation that has sparked calls to action [14,15]. There is a need to gather information from global child health actors in Germany. It is important to be informed about their views and activities in order to understand the reasons for this situation and to assess the role of GCH in Germany’s higher education sector.

This formative research aims to offer insights into the academic landscape of GCH in Germany. Specifically, it will describe the characteristics of experts in the field, their priorities in research and education and analyse perceived opportunities and barriers to working academically within GCH.

## Methods

The study’s sequential explanatory mixed-methods design uses quantitative followed by qualitative methods to improve the understanding of the subject in its complexity and to facilitate the interpretation of the results [16]. For example, conflicting results from the survey can be clarified and explained during the expert interviews, whereby the interview findings and interpretations validate the survey results. The integration of quantitative and qualitative results is underpinned by a joint thematic approach [17].

### Data collection

The selection criteria for the survey participants were current or past involvement in academic GCH research and teaching in a senior position at a German university. We identified and purposefully selected experts from the boards of the German Society of Tropical Paediatrics and International Child Health (GTP) [18] and the German Academy of Child and Adolescent Medicine (DAKJ) [19]. This was done by screening lists from national conference speakers and their professional contacts. Furthermore, the survey encouraged respondents to identify interested colleagues by snowball sampling; no further criteria were given [20]. The survey included an optional request to be contacted for a qualitative interview held by phone using a cloud-based platform.

The survey contained two open- and 31 closed-ended questions (Supplemental online material-1) using the open-source survey software LimeSurvey^TM^ [21]. Survey questions covered participants’ demographics and information about their occupational and research background. Questions about the areas of interest in global health reflected selected priority thematic areas chosen to inform Germany’s global health strategy [22].

Survey findings informed the framework for the interview questions. The interview topic guide included five questions to generate a narrative impulse (Supplemental online material-2). One author conducted the interviews in German, while a second author observed the interview. The interviews took place online (software Zoom^TM^ audio and video, n = 4), by phone (n = 2) or in-person (n = 1), between April and June 2020, were audio recorded and lasted between 16 and 23 minutes. Recordings were transcribed, translated, and pseudonymised.

### Data analysis

Quantitative data were analysed descriptively. Interview transcripts were analysed using qualitative content analysis with inductive category formation [23,24] using MaxQDA 20 software [25]. The authors continuously discussed the category system, emerging difficulties, and the interpretation of the results to complement the perspectives, to validate chains of argumentation, and to avoid singular readings of the material during the reflective process [26]. Finally, quantitative and qualitative data were summarised, interpreted, and jointly integrated. The definition of GCH as ‘the study and practice of improving child health globally’ [27] guided analysis and interpretation.

## Results

Sixteen survey participants were identified and invited; all responded. The 16 initial survey participants identified five additional experts. One participant completed less than 30% of the survey questions and was excluded. Of the 20 survey participants, seven agreed to participate in qualitative interview. We present the findings by integrating the results from the survey and the interviews, highlighting similarities and differences. The four categories identified were as follows: (1) training and professional orientation; (2) professional realities; (3) networking and advocacy, and (4) barriers.

The 20 participants had a median age of 55 years (IQR 17 years), and five were women (Table 1). All participants held at least a Master’s degree, and 11 held a habilitation degree and were professors or had a different postdoctoral title (Associate Professor, ‘Privatdozent’). They described their professional focus as clinical (n = 9), in teaching and research (n = 7) or in administration (n = 4). Thirteen were medical doctors, and nine specialised in paediatrics. Other specialisations mentioned by participants were global health (n = 10) or public health (n = 2).

**Table 1.** Participants’ characteristics.

	Number (Total N = 20)	Percent (%)
Age (in years)		
35–49	6	30
50–64	9	45
>65	5	25
retired	2	10
Gender		
Female	5	25
Male	15	75
Highest academic degree		
Habilitation*	11	55
PhD	2	10
Master’s	1	5
Doctor medicinae (Dr med)^$^	6	30
Professional focus		
Teaching and research	7	35
Administration	4	20
Clinical work	9	45
Proportion of regular working hours spent on global child health topics^§^		
< 25%	15	75
25 − 50%	1	5
51 − 75%	2	10
> 75%	2	10

*In Germany, universities award this postdoctoral degree to scientists certifying them the ability to become full professors; ^$^Dr med – German academic degree resulting from a dissertation; ^§^Topics were identified based on the definition of global child health as ‘the study and practice of improving child health globally’ [27]

### Training and professional orientation

Interviews revealed participants had trained in Germany as medical doctors and/or for postgraduate training. Most participants advanced in clinical medicine or research before engaging in GCH-related activities. As no specialised educational programme for GCH exists in Germany, some participants reportedly had joined postgraduate public health programmes in other countries for example, in the UK (UK). In addition, participants described opportunities to join research or clinical projects abroad as directional for their careers, although most participants’ workplaces are now at German universities.

“I did my specialist training in Germany, and then I went to work abroad. In between, I did two years of paediatric radiology when ultrasound came up because I could use that abroad [and] in between I did a Master’s in community health and health management in Heidelberg.” Participant 5

“For me, the entry point was The Gambia, so to speak. There were the *** Laboratories there, and I somehow found them by chance after asking around for a long time […] They had the whole infrastructure, and there were these laboratory mentors, people who had worked in this field over decades […], so there was this entire tradition [and] they had already done clinical studies before […] I mean, that shaped my life, that I had this opportunity to work in this context. I don’t know exactly how it is in Germany today, but I imagine it is much more difficult.” Participant 2

### Professional realities

Fifteen participants estimated that they currently spent less than 25% of their working hours on GCH-related activities. This time is spent in humanitarian missions or research and teaching, in low- and middle-income countries or as part of the regular work in Germany.

Most participants (13/20) wanted to extend their current activities to at least one additional area, e.g. teaching (n = 11), research (n = 9), consultancy (n = 9), patient care (n = 8) and science communication (n = 8) within the scope of their current occupation. Clinicians (n = 9), however, frequently wanted to expand outside their primary profession into teaching (7/9), consulting (5/9) and research (4/9).

**Figure 1.**
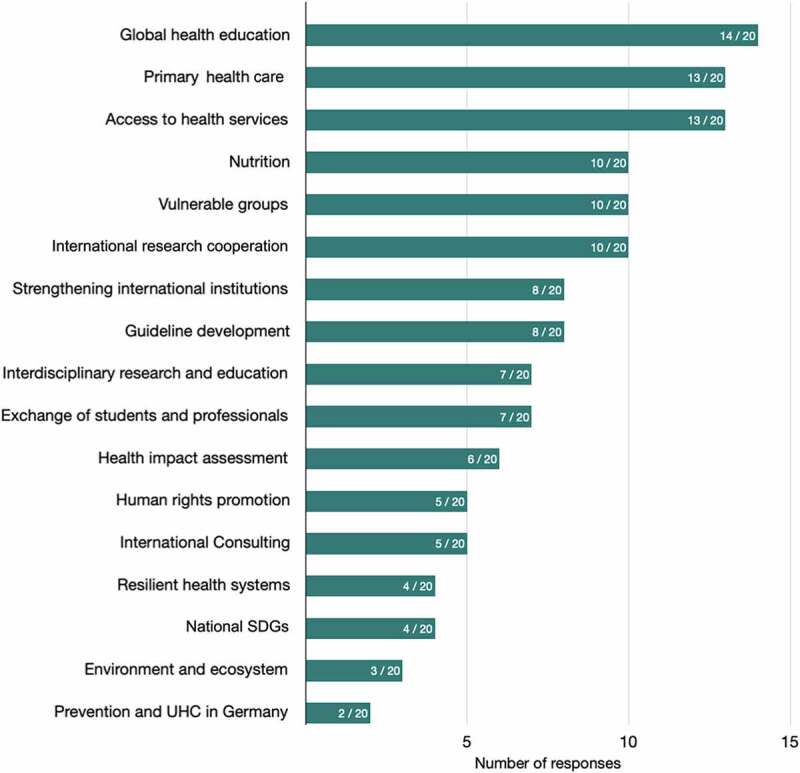
Areas of interest in global health that participants are engaged with. The numbers in the columns indicate the frequency of mentions among the 20 participants. Multiple responses were possible. Eighteen areas of interest were available for selection, assigned to four priority topics according to the position paper prepared by German scientists [22] to inform Germany’s Global Health Strategy [2]: 1. health in all policies (six areas), 2. health systems strengthening (four areas), 3. universal health coverage (four areas), 4. evidence-based care (four areas); SDG- Sustainable Development Goals, UHC- Universal Health Coverage

Participants have a spectrum of areas of interest relevant to GCH they prioritise in their work, such as global health education, primary health care, access to health services, nutrition, vulnerable groups and research cooperation (Figure 1). Participants were less likely to mention areas related to: prevention and universal health coverage in Germany; the environment and ecosystem; work aimed at achieving SDG targets nationally; resilient health systems, and human rights.

Interviews confirmed that most participants follow their interests in GCH in only a fraction of their regular working hours, in which they have too little time for their various interests in the field, and that a majority not only want to extend their activities within their current occupation but also expand and reach out to other occupations. The observation that respondents are keen to invest even more time in GCH underscores the groups’ commitment. Experiences early in individuals’ professional lives and humanitarian motives may fuel this commitment. Retired interviewees remain engaged and follow current debates.

“My main focus? I don’t have any main focus. I do everything. So, of course, I am primarily a paediatrician and clinician […] trying to train people to give better clinical care to patients and teach them techniques like ultrasound. […] the area of prevention or yes, in the broadest sense, vaccinations and nutrition [and] there are a lot of projects going on here to improve care on the ground in Malawi and Tanzania.” Participant 1

“It is rather the question of who is doing global health, including me, that everyone is so busy with it and the additional effort to get more involved or to do more together is simply a question of time and possibilities.” Participant 7

### Networking and advocacy

Participants represented 20 different professional societies and organisations; 11 participants were GTP members. Nineteen survey participants rated the exchange with peers from their field of expertise as important or very important. Furthermore, participants planned to expand their networks internationally with contacts from their field of research (14 vs 1) and inter-professionally (12 vs 2).

For all interviewees, communication was critical for their research and international collaborations. They rely on their own networks as well as those of their organisations. Interviewees felt well networked both in Germany and internationally with researchers from their own discipline and in interdisciplinary projects. On one hand, they were sceptical about the increasing number of online offers and considered personal contacts important. On the other hand, the recent increase in online communication helped consolidate communication with international partners and within the German global health community. They highlighted the annual GTP meeting, the Global Health Hub Germany (GHHG) and the Global Health Alliance Germany (GHA-D) as good networking opportunities. They stressed the need to think outside of their own fields of expertise and to communicate with researchers from other areas such as social scientists, as well as with policymakers and, in international projects, with the local population and experts from other countries.

“We live from networking; we meet at conferences and other events on particular topics. So, we know each other quite well and have formed certain networks. These then also come into play when certain research questions have to be tackled in a superordinate way. And in this respect, I think most of my colleagues, including myself, are relatively well networked.” Participant 4

“In the context of international cooperation, partners with different country and regional experiences also play a role, and it is, of course, a very interdisciplinary field by nature.” Participant 3

**Figure 2.**
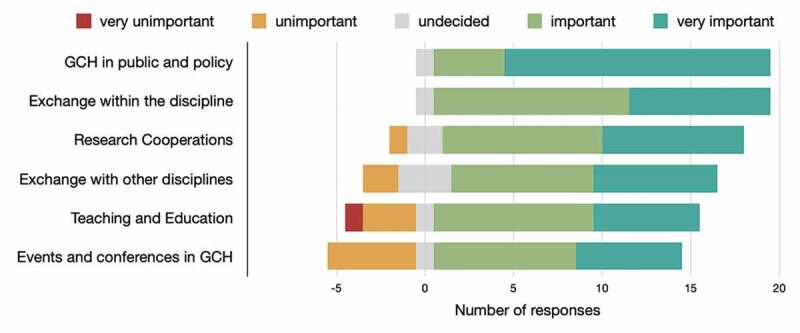
Participants (N = 20) rated means to strengthen GCH in Germany.

Nineteen of the 20 participants expressed that strengthening the societal and political representation of GCH and advocacy were either important or very important, for raising the profile of GCH in Germany and elsewhere. Some respondents viewed increased exchange about teaching topics and more global child health-related events as less important (Figure 2).

“We are a small group anyway, and we have to raise our voice loudly to be heard here in Germany and Europe. And also to have enough impact to be able to intervene in the countries where support is needed. […] We also have the opportunity to network professionally and to gain more influence so that these topics become more present in Germany and not just Covid-19.” Participant 1

“And I imagine that this is also the case in GCH, […] that attempts to exert political influence are necessary to change health care and health services for the benefit of the people, in this case, children and their families. […] Here in Germany, too, actually. But here, it is still difficult to get to it.” Participant 1

### Barriers

Interviewees mentioned structural, personal and historical barriers to the recognition of GCH as a discipline and to their careers specifically. For example, they mentioned some policymakers would prioritise biomedical research in the belief that primarily technologies and medicines can solve global health problems, giving lesser attention to social determinants, health promotion, and prevention and interdisciplinary approaches. Funding opportunities reflect this imbalance and make it harder for global health researchers with such a broader perspective to compete for funds.

“In general, one has to say that there is the problem that less work is done and fewer funds are made available, for example, to strengthen health services in other countries and to improve the capacity of the medical workers there than to look for such magic bullets, some kind of medication that can supposedly solve the problems very quickly.” Participant 6

Few individuals in global health, and particularly in GCH, have leadership positions at German universities. According to interviewees, those few contributed to the recognition and expansion of the field, but could only provide limited representation. Participants were concerned about the common practice of fixed-term contracts, poor recognition in research funding allocation and limited project support. They argue for additional full-time positions to build a more substantial knowledge and human resource base for the entire field and worry about limited representation of GCH topics in the public debate. Most respondents saw their work in global health and GCH as a side-line career, driven by personal commitment and not matched by institutional support.

“But I think what we are missing in Germany is that people really have a job for it and can do it full-time [so] that the potential that we have in Germany can really unfold.” Participant 7

These imbalances have repercussions for the career choices and promotion of young scientists. For example, students and junior doctors show interest in global health and child health, but few opportunities exist for them to advance.

“Basically, we need more structures […] so that these aspects, which are actually in great demand among students, can be transferred into teaching programmes and thus also into research opportunities and research programmes. That is certainly not developed in this way at the moment.” Participant 3

“Precisely because I do a lot of teaching and have a lot to do with students who are asking themselves how they can become and be a doctor and like to do that and realise their interests in global health and justice and things like that. For me, perhaps the most important idea, especially concerning GCH in Germany, is that more opportunities are given to young doctors.” Participant 7

The common practice of fixed-term contracts in global health and GCH occupations was felt to be incompatible with family life.

“My impression is that there are fewer young people who are willing to take risks and actually expect a much more structured life, a structured career […] you have to create something so that young people here get this security and work more in tropical medicine.” Participant 2

Finally, some interviewees compared the GCH landscape in Germany, to the UK and the USA (US). They attributed differences in the research infrastructure to historical roots (e.g. dissimilar colonial histories) and different opportunities for professional exposure abroad.

## Discussion

Our diverse group of respondents appears motivated and enthusiastic about working in GCH. However, their formal employment only allows them to dedicate a fraction of their working hours to the subject. Respondents were keen to engage and progress academically but acknowledged barriers. Given few choices and resources, they tend to focus on distinct areas and prioritise teaching and specific fields such as primary health care and access to health services. They value opportunities for exchange and networking and – despite the barriers – want to expand their activities internationally. In their view, GCH should have more substantial national representation.

The study is the first to provide insights into the academic landscape of GCH in Germany. Furthermore, the analysis of the respondents’ views and perceptions allows hypotheses about the position of GCH within Germany’s higher education sector, which may direct further action to advance GCH in academia.

Our findings indicate a conflict between individuals’ motivation and engagement for GCH, on one hand, and barriers to enter and advance their academic careers in Germany, on the other hand. The group studied is primarily active in research and teaching at German universities and works on a wide range of research topics and projects, mainly in low- and middle-income countries. Especially in research, cooperation with non-medical, social science departments plays a role and is valued. Although respondents’ professional backgrounds and research priorities are diverse, their views on barriers to development in their field of choice, the lack of opportunities for academic progression, representation, and recognition holding back the ambitions of scholars of GCH in Germany, are similar. All of them are aware of and face structural barriers to their academic advancement. Respondents hardly see entry points for careers in GCH, bypassing Germany for postgraduate training or research positions in the UK or the US. So far, only the GTP has established an annual one-week intensive course to prepare paediatricians for service trips abroad which has been running for over 10 years now [28]. Indeed, Germany has been described as a ‘latecomer to the international global health debate’ [3] and its global health research must catch up with other countries [29]. There are few research positions and educational programmes, despite demands by students and faculty [4,5,30,31].

It seems that exceptional motivation and enthusiasm are required to maintain engagement within an adverse academic environment. Nevertheless, maintaining engagement may come with a price. Respondents’ referral to GCH as a side-line academic field, their tendency to focus on niche areas or exclusively work abroad may indicate a certain degree of despair about the situation in Germany. Furthermore, the analysis of the research areas of interest could reveal individuals’ strategies to compensate for the lack of opportunities to progress. For example, respondents hardly mention broader global health topics such as universal health coverage, social determinants of child health or children’s rights. In addition, few respondents use the term ‘GCH’ itself but refer to ‘tropical paediatrics’ and ‘international child health’ instead. Global health is still seeking its identity and multiple definitions exist [32,33]. Nevertheless, these observations may indicate a feeling of a lack of belonging and isolation. What does GCH constitute? Is it a field of study and debate? Is it a sub-discipline? Is it part of paediatrics, global or public health? Alternative explanations for avoiding the term may include a lack of identification with GCH concepts or may simply reflect individuals’ career paths along GCH’s evolution and overlaps with tropical paediatrics, international child and public health.

Respondents focus their research on topics in tropical paediatrics, related to clinical care and services, sometimes in a humanitarian context, and rarely contextualise their work within the wider concepts of GCH and national or international strategies for global health [34]. In addition, they all refer to a single, annual national meeting as a prime opportunity for exchange and networking although a range of more research-focussed, national and international alternatives exist for global health including GCH [35]. There are also few proposals to address the structural career barriers respondents are facing. There is a need for a more in-depth debate within Germany’s child health community about GCH’s scope, role, and position. In contrast to paediatric societies from other countries [36–39], GCH does not feature in Germany’s child health care system [40]. To accept GCH as a new field of study or a sub-discipline, Germany’s academic and professional societies and higher education institutions require GCH experts to formulate a particular research object, underpinning theories, terminologies, and research methods for the support they can provide for funding, titles and positions [41]. Specifically, GCH experts must establish a body of accumulated specialist knowledge, for example, through the scientific evaluation of their educational, clinical, and research activities [27].

The situation described may represent a snapshot in the evolution of an academic entity from infancy to maturity, as has been observed in other fields [42–44], and the barriers GCH is facing may represent opportunities, not dead ends. For example, existing interdisciplinary bonds between nursing sciences, epidemiology, social paediatrics and public health may be instrumental in developing a common definition of GCH accepted across disciplinary boundaries. One could reach out to the disciplines of social sciences, economics, law, and education [45]. Furthermore, paediatric professional and academic societies and funders need to play a more active role. Applying GCH concepts to Germany is another opportunity to gather strengths. Respondents did not prioritise prevention and the implementation of universal health coverage in Germany and the realisation of the national SDGs, probably because of the perceived strengths of Germany’s social security and health financing system [29]. However, children globally and in Germany have been hit hard by the Sars-CoV-2 pandemic [46,47], and COVID-19 threatens to exacerbate child poverty [48]. Advocacy for children’s rights domestically, at the regional and national level, are opportunities for GCH to gain recognition. Such an approach could facilitate the scholarly processing of the universal topics Germany’s global health scientists prioritised [22] with a child health lens and could consolidate the yet dispersed field.

Our formative study has limitations. The study’s sample size was small. Respondents came from professional organisations with specific reference to child health, had affiliations with higher education institutions and had evidence of publication in academic journals and fora. However, researchers relevant to GCH but affiliated with other research organisations not directly linked to paediatrics, may have differing perspectives. Other biases include age and gender. Fourteen of 20 respondents were 50 years of age or older and were predominantly male. The views of younger and female scientists, including students and trainees, would add additional insights. Finally, the study is set in Germany. Research comparing these findings with the situation in other countries is needed to provide valuable perspectives.

## Conclusion

GCH actors in Germany are motivated and engage across professional and academic boundaries. However, without formal affiliations to GCH chairs or institutes at universities, they can afford to give only a fraction of their working hours to their passion. Facing structural barriers to enter or progress in research and education in Germany, they may resort to evasion strategies, confine to niche topics, seek further training or decide to move their career abroad. Respondents are aware of the dilemmas but are uncertain about solutions.

The study offers suggestions for progress. First, GCH actors need to conceptualize GCH, define its role in Germany and beyond; comparing GCH concepts across Europe may be beneficial. Second, the tensions experienced by individuals keen to expand their activities may represent a step on the path to maturity of a new academic sub-discipline, offering opportunities. Third, seizing these opportunities may require reaching out to other disciplines and professions and building alliances to embrace the full spectrum of GCH, including topics such as social determinants and children’s rights, ultimately enabling GCH academics also to engage domestically and apply GCH concepts at home in Germany.

## Data Availability

The quantitative data from the survey that support the findings of this study are available on request from the corresponding author, RW. The qualitative data from the interviews are not publicly available because they contain information that could compromise the privacy of research participants.

## Paper context

Germany’s global health strategy is ambitious and child health globally faces challenges but the universities’ responses appear weak. However, global child health actors are motivated, engage in various topics, value networking opportunities and demand advocacy and stronger representation of the field nationally. They spend only a fraction of their working hours to global child health and want to expand but face multiple barriers to progress. Investment in global child health research and working across disciplines seems necessary to advance the field.
